# Passively Captured Interpersonal Social Interactions and Motion From Smartphones for Predicting Decompensation in Heart Failure: Observational Cohort Study

**DOI:** 10.2196/36972

**Published:** 2022-08-24

**Authors:** Ayse S Cakmak, Erick A Perez Alday, Samuel Densen, Gabriel Najarro, Pratik Rout, Christopher J Rozell, Omer T Inan, Amit J Shah, Gari D Clifford

**Affiliations:** 1 Department of Electrical and Computer Engineering Georgia Institute of Technology Atlanta, GA United States; 2 Department of Biomedical Informatics School of Medicine Emory University Atlanta, GA United States; 3 School of Medicine Emory University Atlanta, GA United States; 4 Emory Healthcare Emory University Atlanta, GA United States; 5 Department of Epidemiology Rollins School of Public Health Emory University Atlanta, GA United States; 6 Atlanta Veterans Affairs Health Care System Atlanta, GA United States; 7 The Wallace H Coulter Department of Biomedical Engineering Georgia Institute of Technology Atlanta, GA United States

**Keywords:** heart failure, mobile device, social interaction, heart disease, mobile health, hospitalization

## Abstract

**Background:**

Heart failure (HF) is a major cause of frequent hospitalization and death. Early detection of HF symptoms using smartphone-based monitoring may reduce adverse events in a low-cost, scalable way.

**Objective:**

We examined the relationship of HF decompensation events with smartphone-based features derived from passively and actively acquired data.

**Methods:**

This was a prospective cohort study in which we monitored HF participants’ social and movement activities using a smartphone app and followed them for clinical events via phone and chart review and classified the encounters as compensated or decompensated by reviewing the provider notes in detail. We extracted motion, location, and social interaction passive features and self-reported quality of life weekly (active) with the short Kansas City Cardiomyopathy Questionnaire (KCCQ-12) survey. We developed and validated an algorithm for classifying decompensated versus compensated clinical encounters (hospitalizations or clinic visits). We evaluated models based on single modality as well as early and late fusion approaches combining patient-reported outcomes and passive smartphone data. We used Shapley additive explanation values to quantify the contribution and impact of each feature to the model.

**Results:**

We evaluated 28 participants with a mean age of 67 years (SD 8), among whom 11% (3/28) were female and 46% (13/28) were Black. We identified 62 compensated and 48 decompensated clinical events from 24 and 22 participants, respectively. The highest area under the precision-recall curve (AUCPr) for classifying decompensation was with a late fusion approach combining KCCQ-12, motion, and social contact features using leave-one-subject-out cross-validation for a 2-day prediction window. It had an AUCPr of 0.80, with an area under the receiver operator curve (AUC) of 0.83, a positive predictive value (PPV) of 0.73, a sensitivity of 0.77, and a specificity of 0.88 for a 2-day prediction window. Similarly, the 4-day window model had an AUC of 0.82, an AUCPr of 0.69, a PPV of 0.62, a sensitivity of 0.68, and a specificity of 0.87. Passive social data provided some of the most informative features, with fewer calls of longer duration associating with a higher probability of future HF decompensation.

**Conclusions:**

Smartphone-based data that includes both passive monitoring and actively collected surveys may provide important behavioral and functional health information on HF status in advance of clinical visits. This proof-of-concept study, although small, offers important insight into the social and behavioral determinants of health and the feasibility of using smartphone-based monitoring in this population. Our strong results are comparable to those of more active and expensive monitoring approaches, and underscore the need for larger studies to understand the clinical significance of this monitoring method.

## Introduction

Although there are numerous attempts to monitor heart failure (HF) in an outpatient setting using wearables and other point-of-care devices, compliance is often an issue and prevents monitoring for extended periods [1,2]. One key sensor system many of us carry with us on a day-to-day basis is the smartphone, and this has been shown to lead to longer patient engagement times than has wearables [3]. In this pilot study, we hypothesized that we could leverage the data recorded on the personal smartphones used by a population with HF to predict decompensation.

We defined HF decompensation status based on worsened functional symptoms or physical examination findings suggestive of lower cardiac output or increased intracardiac pressures. This includes but is not limited to fatigue, dyspnea, hypotension, and lower extremity edema [4]. Treatment includes diuretics and vasodilators intended to improve volume status and cardiac function. Unfortunately, even following successful treatment and return to the euvolemic (normal volume status) state, decompensation episodes can continue to occur with increasing frequency [4,5]. Patil et al [6] reported that about 20% of the patient cohort were readmitted within 30 days of initial hospitalization due to HF, with a median readmission time of 12 days. Furthermore, patients with a lower income had a higher readmission rate, indicating that socioeconomic factors could also contribute to the disease’s progression. If low-cost monitoring methods identify decompensation episodes developing outside the clinic, medical interventions could be administered proactively to prevent hospitalization or other adverse outcomes.

Various studies have investigated techniques for nonintrusively monitoring patients with HF. Packer et al [7] showed that using a combination of clinical variables and impedance cardiography features could be a predictor of a decompensation event in the following 14 days. Previous studies have also investigated the use of wearable devices adhered to the chest. In the “Multisensor Monitoring in Congestive Heart Failure” study [8], the authors propose an algorithm that uses physiological signals, reporting a sensitivity of 63% and a specificity of 92%. However, the authors provide few details and claim it is “proprietary.” Inan et al [9] recorded seismocardiogram signals with a noninvasive wearable patch before and after a 6-minute walk test to analyze the cardiac response to exercise. The authors used graph similarity scores between the rest and recovery phases and found a significant difference between compensated and decompensated groups. In another example, similarity-based modeling was used with physiological signals from a patch on the chest to detect changes from the baseline. This algorithm had a sensitivity of 76% to 88% and a specificity of 85% [10]. Using ballistocardiogram data recorded at home was also investigated [11], and authors demonstrated that collecting high-quality ballistocardiogram data at home is feasible, and an area under the curve of the receiver operator curve (AUC) of 0.78 could be achieved for classifying clinical status.

Other noninvasive approaches include patient-reported outcomes, which could be collected using clinically validated questionnaires such as the Kansas City Cardiomyopathy Questionnaire (KCCQ). The KCCQ assesses the quality of life and predicts readmissions and mortality in patients with HF [12]. In a previous study, Flynn et al [13] reported that KCCQ has modest correlations with exercise capacity measured by the 6-minute walk test in a population with HF.

With the advancement of technology, smartphones have become a ubiquitous part of our daily life. For long-term monitoring, using a smartphone could be advantageous to a solution requiring an additional device by reducing the disruption to patients’ normal daily routine. Our research team and collaborators have previously developed the Automated Monitoring of Symptom Severity (AMoSS) app, which is a custom and scalable smartphone-based framework for remote monitoring [14]. Subsequently, we used the passive data from the first 10 participants of this study to estimate the KCCQ surveys collected through the app [15]. The model estimated the KCCQ score with a mean absolute error of 5.7%, providing an entirely passive method of monitoring HF-related quality of life. (The method was passive in the sense that it does not require any active participation by either the patient or clinical staff beyond the everyday use of a mobile phone to monitor activity and behavioral patterns in the background using software.). In subsequent work, motion data were then used to classify decompensation or compensation events [16]. By using a hold-out test randomly sampled from 30% of the events (N=32), the AUC of the classifier was found to be 0.76.

In this study, HF decompensation events were predicted from features derived from passive and active data collected by the smartphone-based framework. Features were extracted from 3 passive data modalities (motion, location, and social interactions) and 1 active (clinical survey data: short KCCQ [KCCQ-12]). Algorithms based on using a single modality and 2 sensor fusion approaches were developed. An analysis of the feature importance in the model is also presented. Finally, a novel late-fusion model that combines the KCCQ-12, motion, and social contact data is proposed.

## Methods

### Data Collection and Ethical Considerations

Earlier research with the AMoSS app [14] was augmented for use in this study. The app passively collected 3D accelerometer data at a 5-Hz sampling frequency on location, clinical surveys, and digital social contact.

#### Ethical Considerations

**Figure 1:**
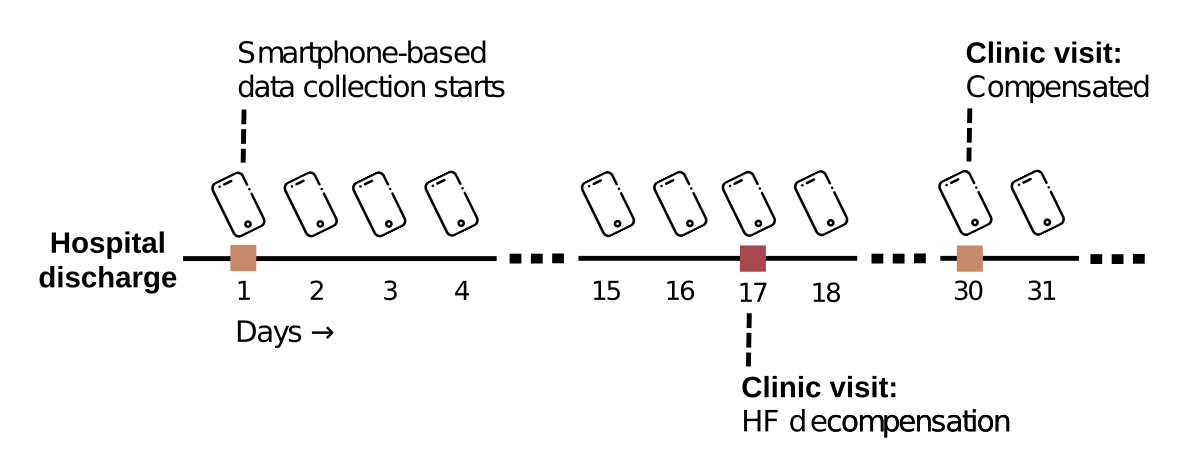
Illustration of the study timeline. Passive data collection started after the hospital discharge, and the clinical team recorded the clinical events after the enrollment. HF: heart failure.

All data were deidentified at the source (on the participants’ phones) with hashed identifiers, and random geographic offsets were added to the location data to protect the participants’ privacy. The data were stored in HIPAA (Health Insurance Portability and Accountability Act)-compliant Amazon Web Services data buckets, and the phone app uploaded data periodically (based on connectivity) every few hours. Participants with HF enrolled in the ongoing study at the Veterans Affairs Medical Center and Emory University Hospital in Atlanta, GA, USA, signed a consent form prior to the beginning of the study. The study protocol was approved by the institutional review board (#00075867) at Emory University. The clinical team provided participants with an Android-based smartphone with the app installed during the enrollment. The participant could opt to stop sharing any data type during the study, using switches provided in the app. Figure 1 illustrates the study timeline after the participant is enrolled. The app passively collected data while the clinical team recorded the clinical events, which consisted of hospital visits with compensated or decompensated status during the enrollment.

#### Data Collection

The data from 28 participants (25 males) who contributed at least 1 clinical event were used in this research. The inclusion criteria for participants in the study were the following: a diagnosis consistent with congestive HF as noted in the electronic medical records within the Emory Health Network, an age over 18 years, the ability to consent to a clinical study, and English as their primary language. Exclusion criteria were the following: diagnosis with a terminal illness with a life expectancy of fewer than 6 months, enrollment in a hospice program, or enrollment in a clinical study that precluded them from participating in another clinical study. Finally, participants had to be willing and able to comply with the use of their smartphones as indicated in the study. Table 1 shows the detailed information about the participants included in the study.

**Table 1:** Data set description: if the metric is not available, the participant is excluded from that row.

Participant characteristics			Values (N=28)
**Sociodemographics**			
	Age (years), mean (SD)		67 (8)
	Male, n (%)		25 (89)
	BMI, mean (SD)		31 (6)
	Mean ejection fraction (%), mean (SD)		35 (17)
	Employed, n (%)		3 (11)
	**Race/ethnicity, n (%)**		
		Black	13 (46)
		White	15 (54)
**Health factors, n (%)**			
	History of diabetes		18 (64)
	Previous myocardial Infarction		2 (7)
	History of hypertension		19 (68)
	Previous stroke		4 (14)
	Peripheral vascular disease		2 (7)
	History of atrial fibrillation		8(29)
	Other non­–atrial fibrillation arrhythmia		3 (1)
**Events**			
	Compensated, n		62
	Decompensated, n		48
	Compensated per person, mean (SD)		2 (1.8)
	Decompensated per person, mean (SD)		2 (1.7)

### Clinical Events

Clinical events consisted of decompensated and compensated events and were collected by the clinical team when the participants visited the hospitals. In the compensated events, the participants visited the hospital for any reason, and their fluid levels were determined to be normal based on the clinician assessment, which includes a history and physical examination. For the decompensated events, the clinical team determined the participant to have functional limitations related to HF. Decompensated and compensated events were assigned to positive and negative classes, respectively.

### Passive Data Sources

**Figure 2:**
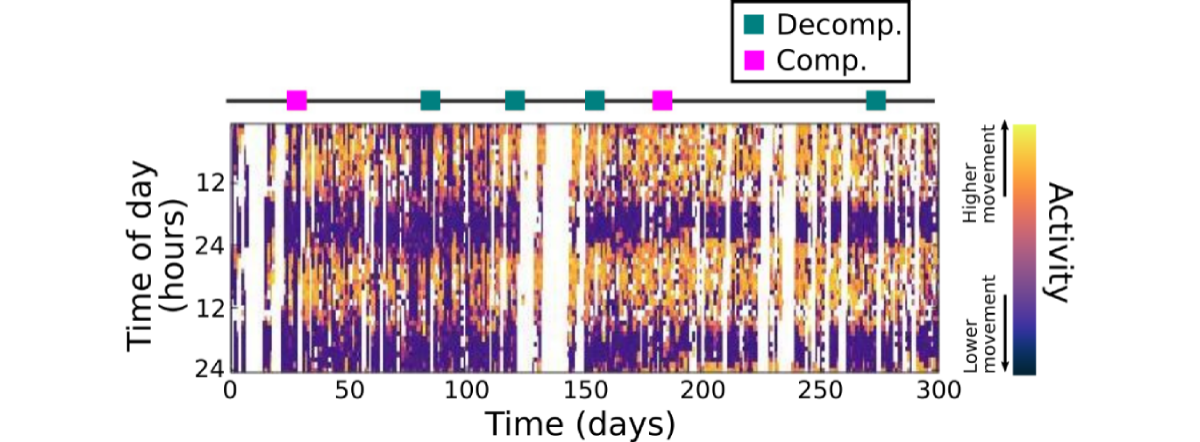
Double plot representation of actigraphy data illustrating daily motion intensity levels for 1 participant. Darker colors indicate lower intensity movement, and the white color indicates missing data. On the top of the plot, decompensated and compensated clinical events are shown with red and orange squares, respectively. Comp: compensated; Decomp: decompensated.

The raw 3D accelerometer data were converted to activity counts using the Actigraphy Toolbox to reduce the required memory for storing [17]. In the first step, the z-axis of the accelerometer data was filtered using a band-pass Butterworth filter with a 0.25 to 11-Hz passband to eliminate extremely slow or fast movements [18]. The maximum values inside 1-second windows were then summed for each 30-second epoch to obtain the activity counts, following a previously described approach [19]. If the participant shared data for less than 0.1% of the analysis window, that window was considered missing. A common way for visualizing motion data in sleep studies to emphasize shifts in sleep rhythms is using a “double plot” format (Figure 2). This figure illustrates the motion data for 1 participant over a recording period of 300 days, and the darker colors indicate lower-intensity movement. Each column consists of 2 consecutive days of data stacked together. The first column shows motion intensity levels on days 1-2, and the second column shows days 2-3, and so on. White regions indicate missing data, which could be due to the participant turning off the data sharing or the smartphone running out of battery.

Social contact data included the contact identifier (ID), directionality, and the duration of each call. Each contact was anonymized and assigned a unique ID at the source (on the phone by the app). The age demographics of our population were such that social media was not uniformly used across the population [20], and therefore, we chose not to capture it to avoid bias. We found that phone calls more so than SMS text messaging were used in our population for digital social interactions. Some participants did not use SMS text messaging at all. We therefore chose to focus on call log data. The phone call log is particularly appealing in an older demographic because it reflects the interactions of close and trusted entities, particularly those that may offer advice on health [21]. Moreover, call logs can be generalized beyond phone calls to any communication medium that is the primary social digital interaction point for close and critical contacts.

**Figure 3:**
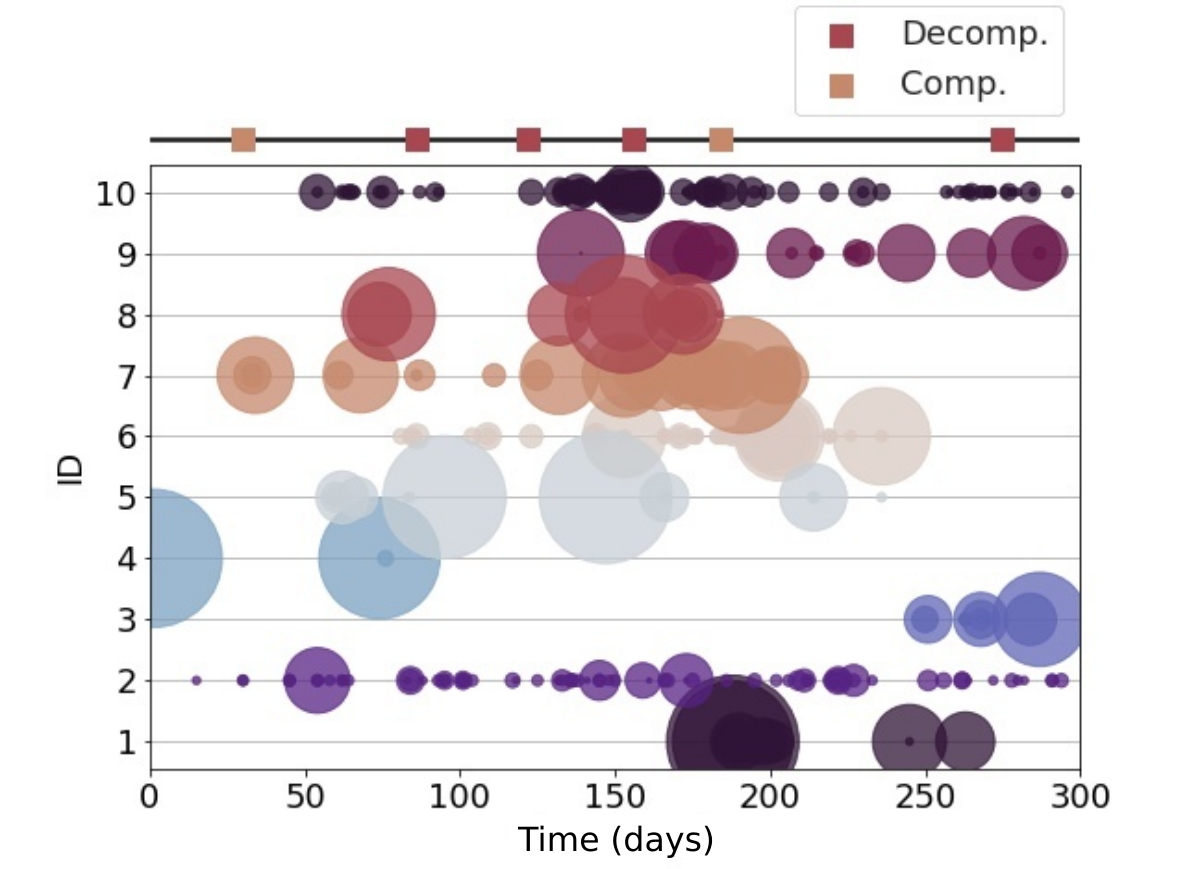
Participants' social contact intensity over 300 days. Each unique contact is assigned a number as shown in the y-axis, and the circle radius is proportional to the call duration to each ID. On the top of the plot, decompensated and compensated clinical events are shown with red and orange squares, respectively. Comp: compensated; Decomp: decompensated.

**Figure 4:**
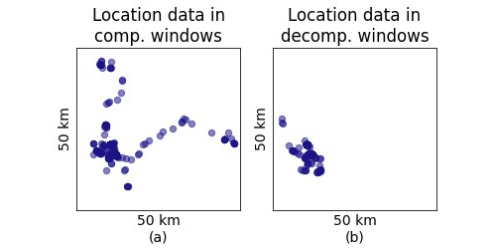
Location data collected in compensated (comp.) and decompensated (decomp.) windows for a participant shown on the same map with 50 km × 50 km dimensions.

**Figure 5:**
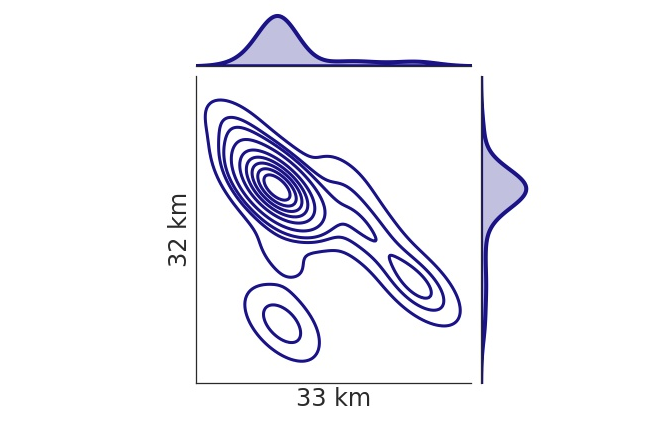
Kernel density estimate for the location data of 1 participant.

Figure 3 illustrates 1 participant’s social contact over 300 days for the 10 most frequently contacted IDs. Lastly, location data were collected using the Android location services app program interface, which generally used cellphone tower or Wi-Fi and not GPS for geolocation. Figure 4 shows the location data of a participant, collected from compensated and decompensated windows. High spatial resolution was not required since the aim was to identify the general environment in which a user was located (eg, home, work, shops). If the smartphone moved at least 100 meters and at least 5 minutes had passed since the last location data update, a new relative location was recorded. These parameters were defined while designing the app to preserve battery life while still providing sufficient temporal and spatial resolution in comparison to the phone’s ability to geolocate without GPS. Figure 5 shows the kernel density estimate of 1 participant’s all-location data updates.

### Active Data Sources

**Figure 6:**
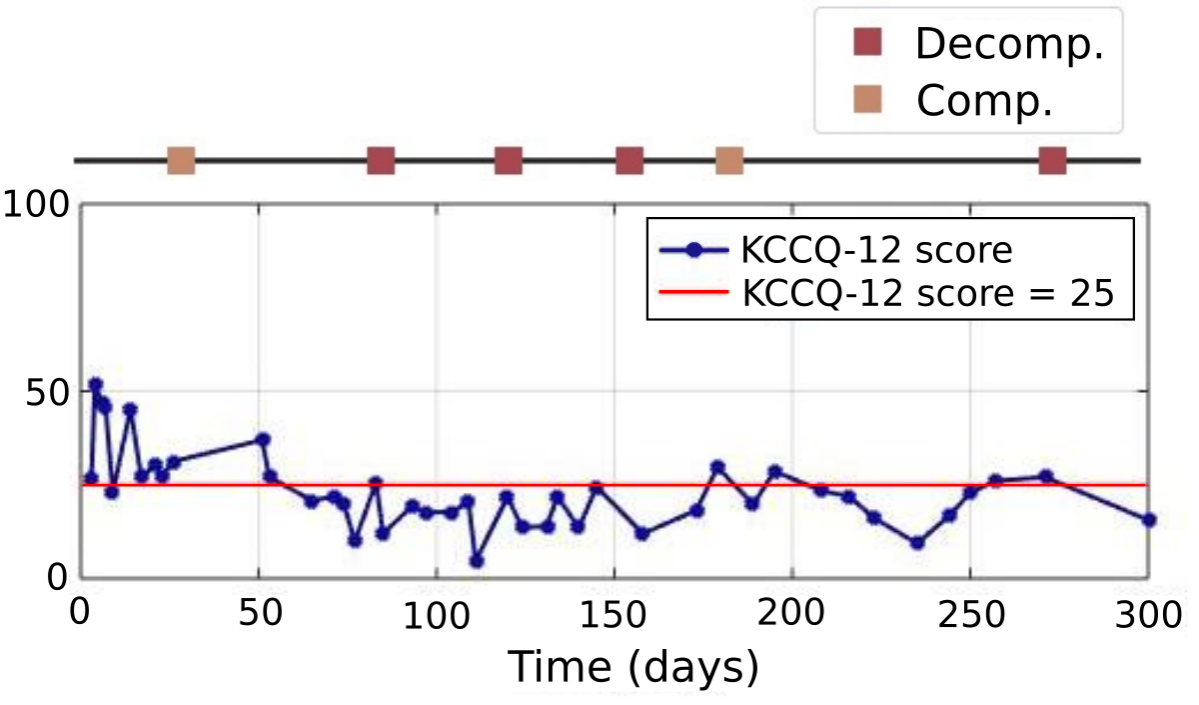
KCCQ-12 summary score over days for a particular participant. A KCCQ-12 score ≤25 indicates a transition to severe HF. Decompensated and compensated clinical events are shown with red and orange squares above the plot, respectively. Comp: compensated; Decomp: decompensated; HF: heart failure; KCCQ-12: short Kansas City Cardiomyopathy Questionnaire.

The active data type, which required user input, was the KCCQ administered through the smartphone app. The scores are lower for severe HF symptoms, and KCCQ scores ≤25 correspond to New York Heart Association class IV. In this study, we used the shorter version of the questionnaire, referred to as the KCCQ-12 [22]. The KCCQ-12 survey had physical limitation, symptom frequency, quality of life, and social limitation domains, and the summary score (ranging from 0 to 100) was the average of all available domains. Figure 6 shows the KCCQ-12 scores administered through the app for a particular participant.

### Feature Extraction and Temporal Windows

Several features were extracted for a particular time window from the data collected through the app to construct the motion feature set. A time window of data was the N day period before a clinical event, and the feature extraction was performed for each time window. The window size N was chosen to be 14 days initially since it was also selected by the developers of KCCQ-12 to represent the participant’s recent functioning [12]. First, from preprocessed smartphone activity counts, descriptive statistics were extracted. These included mean (act_mean_), SD (act_std_), mode (act_mode_), skewness (act_skew_), and kurtosis (act_kurt_). The completeness percentage (act_comp_) was calculated by dividing the epochs with data by the total number of epochs in the time window. For each time window, the total number of calls (*numCalls*), the sum of the duration of calls (*durCalls*), the SD of the duration of calls (*durCalls_std_*), the sum of time without any calls (*durNoCalls*), and the SD of time without any calls (*durNoCalls_std_*) were calculated to be used as social contact features. For these 2 active data feature sets, the performance of using the mean of all surveys inside the window or using the most recent survey was also tested.

Using the participant’s location data, the most frequently visited location was determined and defined as the “home” location. The number of times the participant was at the home location was calculated and used as a feature (*atHome*). For the second location feature, Haversine distances [23] between all locations to the home location were summed (*distToHome*). Finally, the area within a 2-km radius from home was defined as zone 1. The area outside of this radius was defined as zone 2. The number of times the participant contributed from these 2 zones was calculated (*zone 1* and *zone 2*, respectively).

From the KCCQ-12 data, 2 different sets of features were investigated. First, the summation score (*KCCQ-12_sum_*) described in the Active Data Sources section was used as a feature. For the second set of features, each domain (physical limitation, symptom frequency, quality of life, and social limitation) of the KCCQ-12 survey was used separately (*KCCQ-12_all_*).

### Machine Learning Models

Logistic regression classifiers were trained to map the feature vector to the compensated or decompensated outcome. All the models were written in Python 3 language (The Python Software Foundation), and the programming code was based on scikit-learn [24]. Since each participant could contribute to more than 1 event, we used leave-one-subject-out cross-validation. The model was trained on the data from all participants except 1 hold-out participant, and this participant’s data were used as the test set. This process was repeated for each participant in the data set.

Since the number of compensated and decompensated events were highly imbalanced (Table 1), a majority undersampling was performed on the training set before training the classifiers. During the majority undersampling, all participants from the minority class were used, and the same number of participants from the majority class were randomly selected. Sequential forward feature selection was used to select the 3 most informative features from each modality.

**Figure 7:**
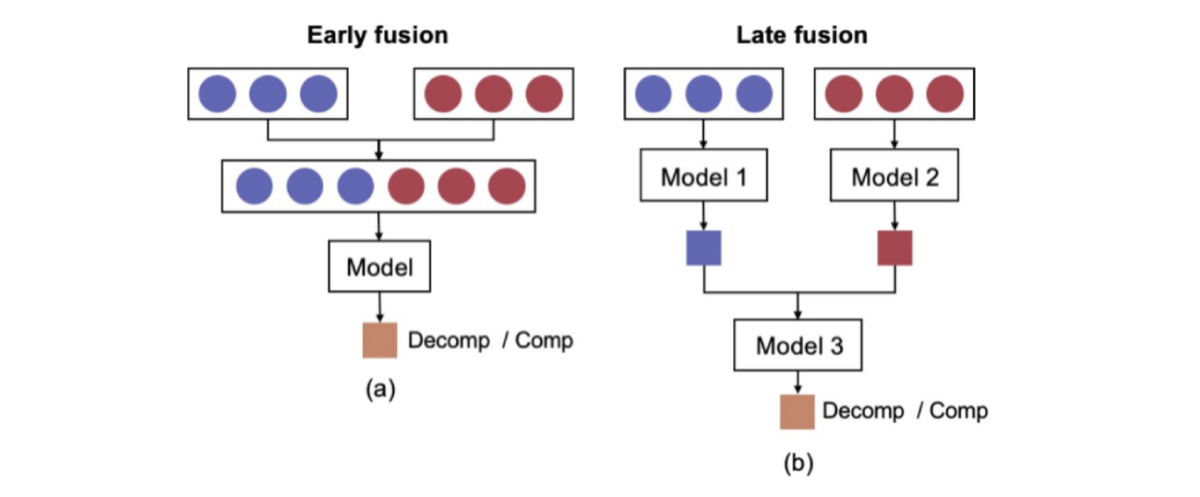
Modality fusion techniques. Purple and red colors indicate 2 different modalities. The left side (a) shows the early fusion approach, and the right side (b) shows the late fusion of the modalities. comp: compensated; decomp: decompensated.

Both early and late fusion approaches combined passive and active modalities (Figure 7). In the early fusion approach, extracted features were combined at the input level of the classifier to create a single feature vector. For the late fusion approach, all single modality models’ output probabilities were concatenated and used as input to another classifier. In all fusion models, the participants who contributed with all data types were included in the analysis. Each analysis was repeated 50 times with different seeds. The mean and SD of the repeats were then presented as results.

To examine and interpret the features further, Shapley additive explanation (SHAP) values for the early fusion model were calculated [25]. This framework is model agnostic, and SHAP values quantify the contribution and impact of each feature to the model.

Finally, we investigated how early the models can predict an outcome by implementing a time-to-event analysis and a window size analysis. The time-to-event methodology consisted of analyzing the performance of a model using data from only 1 day prior to the event but shifting which day is included in the analysis. The window size methodology consisted of analyzing different intervals of days prior to the event and evaluating the model performance on each window.

## Results

### Single Modality Model Results

The cross-validation performance for each single-modality model (motion, location, and social contact) is shown in Table 2. For these experiments, the time window was set to 14 days before each clinical event. The number of unique participants and the number of clinical events changed according to the modality since the participants could stop contributing data. For the motion model, 23 participants contributed with 28 decompensated events and 44 compensated events. For the social contact model, there were 21 participants with 27 decompensated events and 45 compensated events. Finally, there were 18 participants with 13 decompensated events and 33 compensated events for the location model.

**Table 2:** Passive data model performance results presented as the mean and SD of the external folds of each experiment.

Modality	Accuracy, mean (SD)	AUC^a^, mean (SD)	AUCPr^b^, mean (SD)	PPV^c^, mean (SD)	TPR^d^, mean (SD)
Motion	0.66 (0.03)	0.66 (0.03)	0.60 (0.06)	0.55 (0.04)	0.61 (0.06)
Location	0.59 (0.07)	0.56 (0.10)	0.39 (0.11)	0.34 (0.10)	0.49 (0.17)
Social	0.58 (0.05)	0.65 (0.05)	0.56 (0.06)	0.46 (0.06)	0.60 (0.07)

^a^AUC: area under the curve of the receiver operator curve.

^b^AUCPr: area under the precision-recall curve.

^c^PPV: positive predictive value.

^d^TPR: true positive rate.

Table 3 provides the single modality results for the active data type, the KCCQ-12 survey. The table shows the performance metrics when the mean of all the questionnaires within the 14-day window was used and when the most recent questionnaire was used for the 2 different active feature sets (KCCQ-12_sum_ and KCCQ-12_all_). For this active data type, 20 unique IDs contributed with 23 decompensated events and 32 compensated events. Using the summary KCCQ-12 score and taking the most recent questionnaire resulted in the highest area under the precision-recall curve (AUCPr) score of 0.69.

**Table 3:** Active data single modality model performance reported as the mean and SD of the external folds of each experiment.

Modality		Accuracy, mean (SD)	AUC^a^, mean (SD)	AUCPr^b^, mean (SD)	PPV^c^, mean (SD)	TPR^d^, mean (SD)
**Mean of window**						
	KCCQ-12_sum_^e^	0.64 (0.01)	0.75 (0.01)	0.61 (0.02)	0.55 (0.01)	0.66 (0.03)
	KCCQ-12_all_^f^	0.65 (0.02)	0.67 (0.02)	0.54 (0.04)	0.57 (0.02)	0.69 (0.04)
**Most recent**						
	KCCQ-12_sum_	0.69 (0.01)	0.77 (0.01)	0.69 (0.02)	0.61 (0.02)	0.71 (0.03)
	KCCQ-12_all_	0.69 (0.03)	0.70 (0.01)	0.61 (0.04)	0.60 (0.02)	0.74 (0.04)

^a^AUC: area under the curve of the receiver operator curve.

^b^AUCPr: area under the precision-recall curve.

^c^PPV: positive predictive value.

^d^TPR: true positive rate.

^e^KCCQ-12_all_: set of features for each short Kansas City Cardiomyopathy Questionnaire survey domain separately.

^f^KCCQ-12_sum_: summation scores for all short Kansas City Cardiomyopathy Questionnaire survey domains.

### Fusion Modality Model Results

For the fusion model which combines KCCQ-12 and motion data, 17 participants contributed data for both modalities, with 21 decompensated events and 26 compensated events. When 3 modalities were used (KCCQ-12, motion, and social contact), 16 participants contributed with 18 decompensated events and 21 compensated events. Finally, when all data types were merged (KCCQ-12, motion, social contact, and location), there were data available for 12 participants, with 10 decompensated events and 18 compensated events.

**Figure 8:**
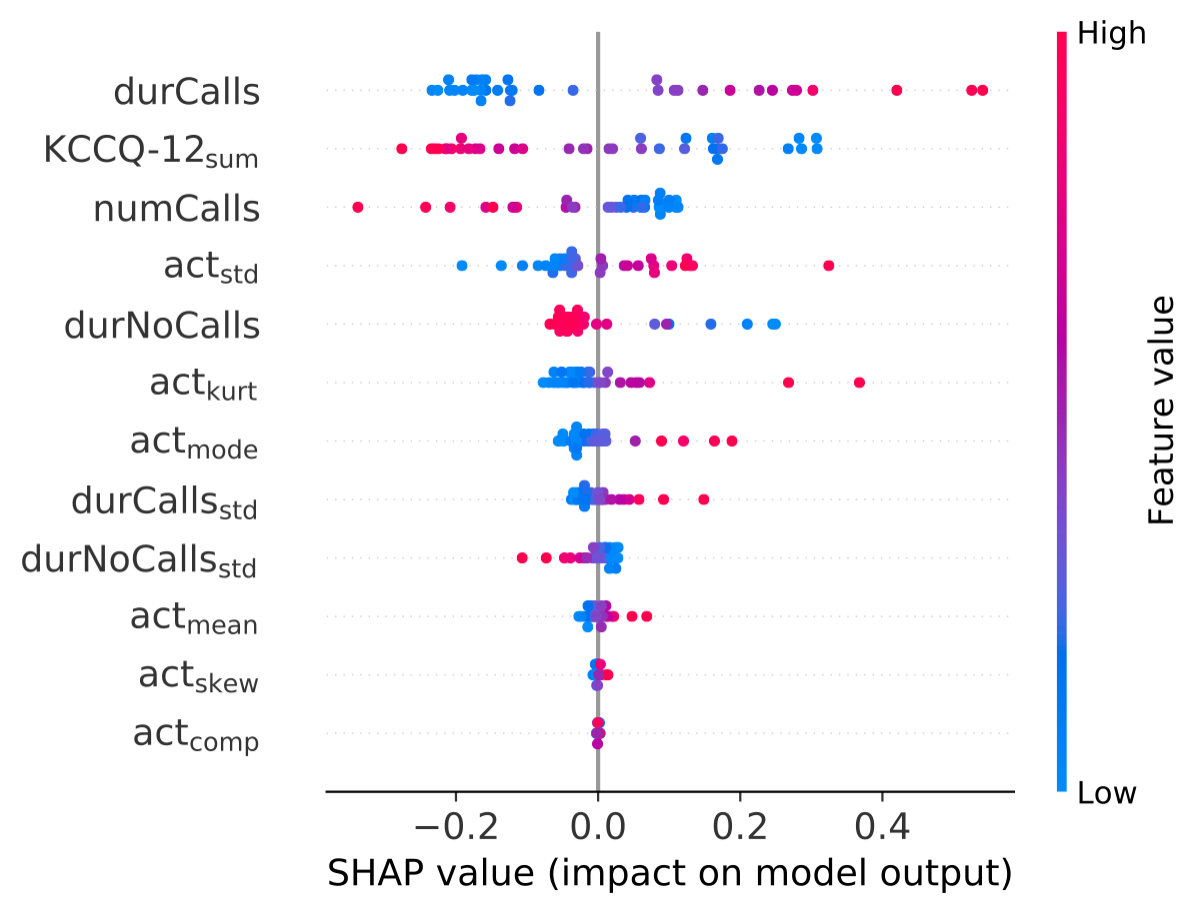
SHAP summary plot for the early fusion model. Features are sorted by their impact on the y-axis. Each point on the plot shows the Shapley value for 1 instance. The horizontal location shows the feature’s effect for predicting positive class (decompensated) or negative class (compensated), and color indicates the feature value. SHAP: Shapley additive explanation.

The results for the early fusion models are shown in Table 4. For the late fusion models, the results are shown in Table 5. The highest AUCPr of 0.77 was achieved when KCCQ-12, motion, and social contact modalities were combined with late fusion. For the early fusion models, using the same modalities resulted in an AUCPr of 0.69. The corresponding SHAP summary plot for the early fusion model is shown in Figure 8.

**Table 4:** Results of early fusion models reported as the mean and SD of the external folds of each experiment.

Modality	Accuracy, mean (SD)	AUC^a^, mean (SD)	AUCPr^b^, mean (SD)	PPV^c^, mean (SD)	TPR^d^, mean (SD)
Motion + social	0.62 (0.04)	0.58 (0.03)	0.54 (0.04)	0.53 (0.05)	0.53 (0.06)
KCCQ-12^e^ + motion	0.73 (0.02)	0.81 (0.01)	0.75 (0.03)	0.69 (0.02)	0.73 (0.05)
KCCQ-12 + motion + social	0.71 (0.04)	0.72 (0.05)	0.69 (0.06)	0.70 (0.04)	0.66 (0.09)
KCCQ-12 + motion + social + location	0.67 (0.05)	0.64 (0.07)	0.57 (0.11)	0.55 (0.07)	0.56 (0.09)

^a^AUC: area under the curve of the receiver operator curve.

^b^AUCPr: area under the precision-recall curve.

^c^PPV: positive predictive value.

^d^TPR: true positive rate

^e^KCCQ-12: the short Kansas City Cardiomyopathy Questionnaire survey.

**Table 5:** Results of late fusion models reported as the mean and SD of the external folds of each experiment.

Modality	Accuracy, mean (SD)	AUC^a^, mean (SD)	AUCPr^b^, mean (SD)	PPV^c^, mean (SD)	TPR^d^, mean (SD)
Motion + social	0.64 (0.03)	0.63 (0.04)	0.52 (0.05)	0.54 (0.04)	0.56 (0.07)
KCCQ-12^e^ + motion	0.67 (0.03)	0.75 (0.02)	0.67 (0.04)	0.61 (0.03)	0.72 (0.07)
KCCQ-12 + motion + social	0.71 (0.04)	0.79 (0.03)	0.77 (0.04)	0.68 (0.04)	0.70 (0.05)
KCCQ-12 + motion + social + location	0.62 (0.07)	0.72 (0.07)	0.60 (0.11)	0.49 (0.07)	0.68 (0.10)

^a^AUC: area under the curve of the receiver operator curve.

^b^AUCPr: area under the precision-recall curve.

^c^PPV: positive predictive value.

^d^TPR: true positive rate.

^e^KCCQ-12: the short Kansas City Cardiomyopathy Questionnaire survey.

### Time-to-Event and Window Size Analysis

We investigated how early the algorithms can predict an outcome by shifting the days to the event and using different window sizes in days for each model in each category.

**Figure 9:**
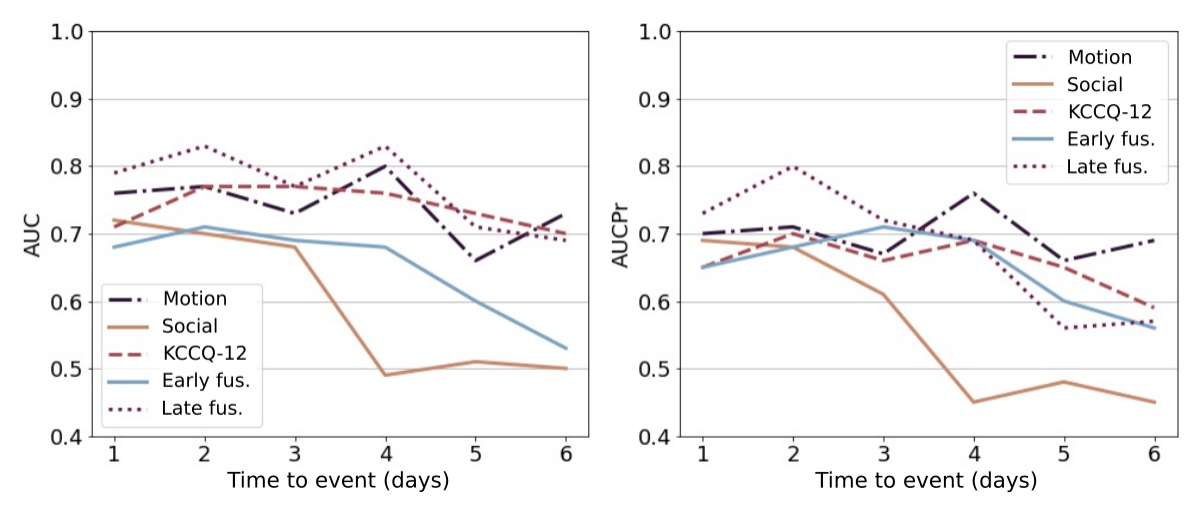
Performance changes as the days to events are shifted. The x-axis indicates the time to event in days, and the y-axis indicates the AUC and AUCPr performance. Early fusion and late fusion models combine KCCQ-12, motion, and social contact modalities. AUC: area under the curve of the receiver operator curve; AUCPr: area under the precision-recall curve; fus: fusion; KCCQ-12: the shot Kansas City Cardiomyopathy Questionnaire.

Figure 9 illustrates the AUC and AUCPr change of each model as the time in days to the event is increased. Only participants who contributed data during the time-to-event intervals and event type were included (n=13; with 13 decompensated events and 18 compensated events). We observed a decrease in performance on the social contact modality when the time to event was 4 days. However, the motion model performance peaked at 4 days to the event. The best model was the late fusion model with a prediction window of 2 days prior to the event (Figure 9). This best model had an AUC of 0.83, an AUCPr of 0.80, a positive predictive value (PPV) of 0.73, a sensitivity of 0.77, and a specificity of 0.88. The 4-days-ahead model had a similar but lower performance with an AUC of 0.82, a AUCPr of 0.69, a PPV of 0.62, a sensitivity of 0.68, and a specificity of 0.87.

**Figure 10:**
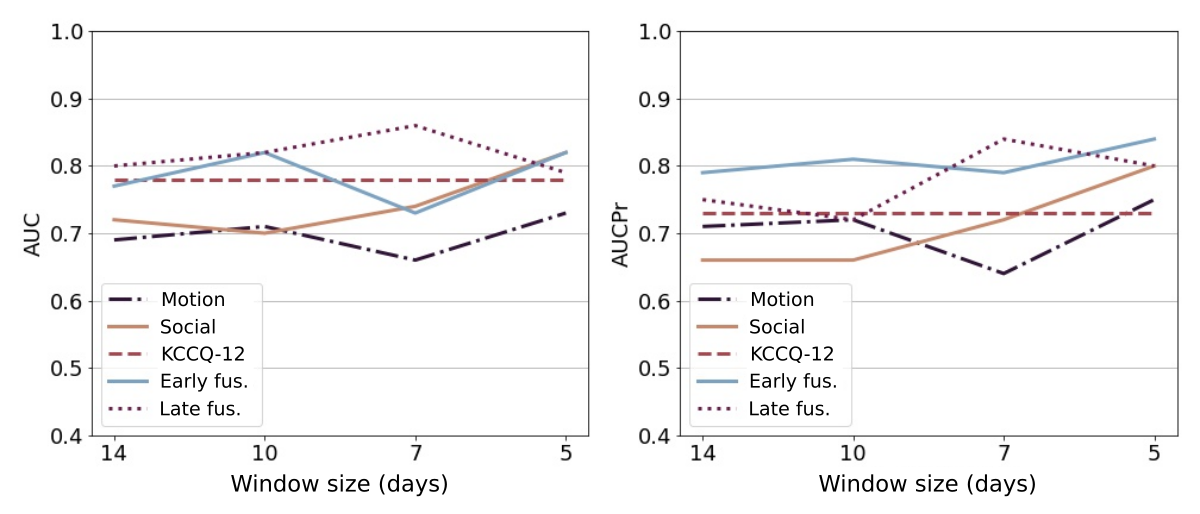
Performance changes as the window size is reduced. The x-axis indicates the time to event in days and the y-axis indicates the AUC and AUCPr performance. Early and late fusion models use KCCQ-12, motion, and social contact modalities. AUC: area under the curve of the receiver operator curve; AUCPr: area under the precision-recall curve; fus: fusion; KCCQ-12: the shot Kansas City Cardiomyopathy Questionnaire; win: window.

Figure 10 illustrates the performance of the models when the window size is increased. Participants with all the window size data and event type were included (n=11; 12 decompensation events and 15 compensation events). We observed that the performance of the KCCQ-12 model was similar across all window sizes. However, the performance of the social contact model improved as the window size decreased.

## Discussion

### Overview

In this proof-of-concept study that involved tracking HF status with smartphone technologies, we showed that it is feasible to collect information from self-reported surveys and passive monitoring that are clinically relevant in classifying compensated versus decompensated status. This study is a first of its kind to evaluate 3 passive data modalities (motion, location, and social interactions) and 1 active data modality, the KCCQ-12 survey. We tested both individual and combined active and passive metrics, and showed that each of them individually and in combination may be potentially useful in helping predict HF decompensation up to 6 days in advance of the clinical encounter.

### Principal Findings

Next-day prediction algorithms were built using each modality separately. From the passive data sources, the motion data–based model achieved the highest AUCPr of 0.60. For a model based only on the responses of the KCCQ-12, using the summary of all domains and using the most recent score resulted in the best performance with an AUCPr of 0.74 (Table 3). Combining both passive and active data modalities achieved a superior performance compared to models based on passive or actively collected data alone (see Tables 4 and 5). The highest performing model combined KCCQ-12, motion, and social contact data. Using the late fusion approach achieved a 6% higher AUCPr compared to early fusion when 3 modalities were used. Late fusion summarizes each modality and presents a lower-dimensional vector to the final classifier [26]. Therefore, this method could reduce the chances of overfitting and addresses the curse of dimensionality when the sample size is small. An AUC of 0.83, an AUCPr of 0.80, a PPV of 0.73, a sensitivity of 0.77, and a specificity of 0.88 for this model may indicate that the approach could potentially add clinical interventions into the framework and result in a low number of false alarms.

Figure 8 illustrates the feature importance using the SHAP method. Duration and number of calls were among the most informative features, indicating that the dynamics of social interactions could be affected by the disease status. The SHAP summary plot also indicates that a higher duration but fewer calls results in a higher probability of HF decompensation for the model. Another important feature was the KCCQ-12 summary value, and a lower value of this parameter gave rise to higher SHAP values. The SHAP plot also indicated that higher mean smartphone motion intensity resulted in a higher probability of HF, which was unexpected since HF limits daily physical activity and is often associated with fatigue.

When different time-to-event horizons were tested, a general trend of lower performance for longer future predictions was observed. This was expected since symptoms are likely to become more pronounced closer to the event. However, predictions 2 days ahead were actually better than those 1 day ahead, and the performance 4 days ahead was almost as good as that 1 day before the event. This indicates that 1-day, 2-day, and 4-day models could be run simultaneously to identify short- and medium-term risks and result in different levels of intervention. Changes in performance will be affected by the levels of missingness as the event approaches, as well as the intrinsic behaviors, which may explain the performance of the 2-day window.

### Comparison With Other Work

Our proof-of-concept study suggests that low-burden, smartphone-based methods of monitoring in HF may offer modest incremental predictive value. The accuracy of our models was similar to earlier work that used mobile health sensors [10] although the lead time was less. We obtained similar results with a late fusion model with a sensitivity of 77% and a specificity of 88% two days prior to the event. However, only a modest reduction in performance was seen for a 4-day prediction window, particularly using motion only, suggesting that running multiple models for different prediction windows may be appropriate. Similarly, the Link-HF study reports a sensitivity of 76% to 88% and a specificity of 85% in a median time of 6.5 (IQR 4.2-13.7) days prior to HF readmission [10]. Although the lead time is lower in our study (2 vs 6.5 days), the costs and burden are lower as well. Two-day advanced alerts may still accelerate care and trigger earlier treatments than may usual care although more research is needed. Any reduction in delays of care with proactive monitoring and intervention may reduce the overall HF burden; nonetheless, the impact on costs and mortality remain to be explored. Because this is the first study of its kind, our primary focus was on the discovery of novel social and behavioral metrics that help to understand the biopsychosocial mechanisms underlying HF. As such, it underscores the need for larger studies aimed at training and testing models with larger lead times and the potential to reduce HF readmissions with sufficient statistical power.

### Limitations

There are several key limitations to this study. First, when the data were missing, the app did not indicate whether this resulted from the participant closing the app voluntarily or if it resulted from the smartphone battery running out. These behaviors have different etiologies, which may be related to impending decompensation in different ways. For example, closing the app may indicate being tired, whereas a battery running out of charge may indicate apathy connected with depression. If an additional label is collected for missing sections, it could be used to learn other behavioral patterns. Second, text messages and social media can provide a more complete picture on social contact. However, due to the age demographics of our population, social contact was quantified using only phone call information [20]. Despite the limited data, our results showed a strong association with decompensated HF status and phone call information. Third, even though each participant contributed many days, the study’s sample size was relatively small (N=28 participants), and, therefore, the methods should be further validated in a larger cohort. Finally, the reliance on hospital records rather than on independent examination of participants might have led to misclassification. We cannot rule out the possibility of unmeasured confounders in those who did and did not experience decompensation events, and our limited sample size restricted our ability to examine this as well. The small sample size also restricted our ability to examine differences by age and HF severity. Nevertheless, we were able to show the feasibility of combining passive and active features extracted from a mobile device to predict HF events. Our findings provide good evidence that we should perform a larger confirmatory study.

### Conclusions

Our proposed novel smartphone-based approach for noninvasively monitoring patients with HF may help monitor health status changes through changes in movement, location, social interactions, or a combination of these. Many of these features are new discoveries and suggest important mechanisms of disease that have previously been less explored. Due to the ubiquity of smartphones and the ease of scalability of the framework, our method has the potential to facilitate low-cost monitoring of large populations. However, we note that this is a preliminary study on a relatively small population, and before it can be validated, a larger study is required. In addition, other passive monitoring devices (such as movement sensors in the house, electricity usage monitors, and home alarm systems) may provide additional useful information on the changes in behavior leading up to an intervenable event. Moreover, in future work, the feasibility of combining the proposed method with clinical interventions (such as teleconsultations and drug dose modification) will need to be investigated to measure the potential impact of the framework described in this paper.

## Abbreviations

act_comp_: completeness percentage activity counts
act_mean_: mean of activity counts
act_mode_: mode of activity counts
act_kurt_: kurtosis activity counts
act_skew_: skewness activity counts
act_std_: SD of activity counts
AMoSS: Automated Monitoring of Symptom Severity
atHome: number of times the participant was at the home location
AUC: area under the curve of the receiver operator curve
AUCPr: area under the precision-recall curve
distToHome: sum of Haversine distances between all locations to the home location.
durCalls: sum of the duration of calls
durCalls_std_: SD of the duration of calls
durNoCalls: sum of time without any calls
durNoCalls_std_: SD of the time without any calls
HF: heart failure
HIPAA: Health Insurance Portability and Accountability Act
KCCQ: Kansas City Cardiomyopathy Questionnaire
KCCQ-12: short Kansas City Cardiomyopathy Questionnaire
KCCQ-12_all_: set of features for each KCCQ-12 survey domains separately
KCCQ-12_sum_: summation scores for all KCCQ-12 survey domains
NHLBI: National Heart, Lung, and Blood Institute
NIH: National Institutes of Health
numCalls: total number of calls
PPV: positive predictive value
SHAP: Shapley additive explanation
TPR: true positive rate
zone 1: number of times the participant was within a 2-km radius from home
zone 2: number of time the participant was outside the 2-km radius from home
